# Long-term survival of a patient with microsatellite-stable refractory colorectal cancer with regorafenib and PD-1 inhibitor sintilimab: a case report and review of literature

**DOI:** 10.1186/s12876-021-01950-y

**Published:** 2021-10-23

**Authors:** Yong Zhang, Fang Zhang, Lingdi Zhao, Xiaomin Fu, Yiman Shang, Quanli Gao

**Affiliations:** Department of Immunotherapy, Affiliated Cancer Hospital of Zhengzhou University and Henan Cancer Hospital, No 127, Dongming Road, Jinshui District, Zhengzhou City, 450003 Henan Province China

**Keywords:** Colorectal cancer, Microsatellite, Regorafenib, PD-1 inhibitor, Sintilimab

## Abstract

**Background:**

Colorectal cancer (CRC) is the third most prevalent cancer worldwide and poses a serious challenge for clinicians. Previous studies have shown promising results in patients with Microsatellite Stable microsatellite-stable CRC refractory to chemotherapy upon treating with (Programmed Cell Death Protein 1) PD-1 inhibitor combined with regorafenib. Herein, we report a unique case of a patient for whom the conventional chemotherapy and radiotherapy were ineffective, but showed a prolonged stable disease with third-line treatment with regorafenib and PD-1 inhibitor, sintilimab.

**Case presentation:**

A 64-year-old East Asian female patient was admitted to a regional cancer hospital presenting with abdominal unease due to increased stool frequency and bloody stool. Digital anal examination revealed adenocarcinoma, while genetic profiling of the tumor resections detected wild-type KRAS mutations in codon 12 and 13. Microsatellite instability (MSI) analysis for detecting germline mutations of (Mismatch-repair) MMR genes showed stable phenotype. In December 2016, Miles’ resection for intestinal adhesion release and iliac vessel exploration in the rectum was performed (Tumor, Node, Metastasis [TNM]: T3N0M0; stage IIA). The adjuvant chemotherapeutic regimen consisted of a combination of capecitabine at 1.5 g (twice daily) and oxaliplatin therapy at 200 mg for three cycles from February 2016; followed by administering capecitabine tablets orally (1.5 g bid) for five cycles as post-operative palliative care. The patient tested positive for hepatic C virus, which was managed by oral antiviral agents. Following recurrence of rectal adenocarcinoma after 4 years and disease progression with a previous chemotherapeutic regimen, regorafenib was administered at 120 mg once daily combined with sintilimab 200 mg, and the patient's progress was monitored. A follow-up computerized tomography imaging in March 2020 showed disease progression, additionally presented nodule formation (TNM: T3NxM1b; stage IVB). According to Response Evaluation Criteria in Solid Tumors criteria (RECIST), the patient showed a complete response (CR) after treatment with regorafenib and sintilimab immunotherapy.

**Conclusion:**

Data from this clinical case report support future exploration of combination treatment of the oral multi-kinase inhibitor regorafenib with PD-1 targeted monoclonal antibodies in patients with metastatic microsatellite-stable CRC.

**Supplementary Information:**

The online version contains supplementary material available at 10.1186/s12876-021-01950-y.

## Background

According to the GLOBOCAN 2020 data, colorectal cancer (CRC) constitutes about 10.6% of the total number of new cases in 2020 [1]. Despite the significant improvement in treatment approaches, it causes considerable mortality and morbidity in men and women [2]. CRC develops due to several biochemical processes that are modulated by genetic mutations, microenvironment factors, and epigenetic alterations such as microRNAs (miRNAs) [3]. Hence, the role of miRNAs, mast cells, Kirsten Rat Sarcoma (KRAS) and v-raf murine sarcoma viral oncogene homologue B (BRAF) have been explored as potential biomarkers for CRC [4, 5]. The two most important pathways involved in colorectal carcinogenesis are the epidermal growth factor receptor (EGFR) signaling pathway comprising KRAS and BRAF mutations and the DNA mismatch-repair system [6]. The KRAS codon 12 and 13 mutations cause constitutive activation of the KRAS protein by revoking guanosine triphosphatase (GTPase) activity. Antibodies targeting the EGFR might be ineffective against the unregulated downstream signaling generated by these mutations [7], although their benefit is confined to KRAS wild-type tumors only [8].

Immune checkpoint inhibitors (ICIs) are currently under investigation as a treatment option for patients with CRC [2, 9]. Recent advances in molecular genotyping have demonstrated that CRC with the subset of mismatch-repair-deficient or microsatellite instability-high (dMMR/MSI-H) tumors are most likely to respond with immunotherapeutic agents [10]. However, immunotherapy alone provides minimal clinical benefit to the smaller subset of CRC with microsatellite-stable (MSS) tumors [11]. ICIs are being explored as an alternative treatment option in this specific cohort as well.

Pembrolizumab (Keytruda) and Nivolumab (Opdivo®) are the two recognized programmed death-1 (PD-1) inhibitors approved by the Food and Drug Administration (FDA) for patients with metastatic CRC with dMMR or MSI-H [12]. A recent discovery of another fully humanized monoclonal antibody, ipilimumab (Yervoy®), was approved by the FDA for use in combination therapy with nivolumab in patients with dMMR refractory to a previous chemotherapeutic regimen [13]. In addition, sintilimab (Tyvyt®), also a monoclonal antibody against PD-1, which works by blocking the association between PD-1 and its ligands, has been approved by the National Medical Products Administration (NMPA) of China to treat relapsed or refractory classical Hodgkin lymphoma in patients refractory to two or more lines of systemic chemotherapy [14]. The clinical studies of pembrolizumab and the ipilimumab-nivolumab combination have shown that patients develop immune resistance to the intervention in due course [13].

Contextually, the administration of PD-1 inhibitors as monotherapy is debatable in MSS patients with CRC. In a landmark development, a clinical trial study in a cohort of 24 Japanese patients with MSS chemoresistant metastatic colorectal cancers, administration of regorafenib and nivolumab reported a response rate of 33% and a median progression-free survival of > 6 months [15]. These promising results from the Regorafenib Plus Nivolumab (REGNIVO) trial [15] suggested a potential clinical benefit for patients with MSS CRCs refractory to chemotherapy treated with PD-1 inhibitor combined with regorafenib.

Based on this rationale, herein, we report a unique case study of a patient with recurrent MSS CRC with KRAS wild-type mutations, refractory to oxaliplatin‐ and irinotecan‐based chemotherapy combined with bevacizumab, who demonstrated a stable disease to regorafenib and sintilimab combination treatment. We aim to highlight the encouraging antitumor activity of combining regorafenib and sintilimab as a third-line treatment option for CRC refractory to first- and second-line treatments.

## Case presentation

A 64-year-old Chinese female patient admitted to a tertiary care cancer hospital was presented with abdominal unease due to increased stool frequency and bloody stools. Digital rectal examination revealed adenocarcinoma, computerized tomography (CT) imaging identified inguinal nodule shadow and enlarged lymph nodes, which implied the presence of rectal cancer. Further, a mediastinal aortic arch nodule shadow indicated metastasis. Subsequent histopathological examination of the biopsy confirmed the rectal adenocarcinoma. The patient got admitted to the general surgery department in January 2016 and underwent Miles’ resection to remove the distal portion of the pelvic colon, followed by intestinal adhesion release surgery that relieved the intestinal obstruction. Iliac vessel exploration facilitated containment of the hemorrhagic episodes. Post-operative histopathological results reported ulcerative and moderately differentiated adenocarcinoma (in the rectum and part of the sigmoid colon) with the invasion of adventitia. Based on the American Joint Committee on Cancer TNM classification system, the tumor stage was T3, N0, M0 (IIA). Genetic profiling of the tumor resections detected wild-type KRAS codon 12 and 13 mutations. Also, MSI analysis for detecting germline mutations of MMR genes showed microsatellite-stable (MSS) phenotype. The adjuvant chemotherapy regimen consisted of three cycles of oral capecitabine 1.5-g tablets (twice daily) and oxaliplatin 200 mg from February 2016, followed by four cycles of capecitabine tablets 1.5 g (twice daily). Additional file 1 illustrates a detailed pictorial case presentation. The patient’s medical history included a cesarean section 30 years ago and no familial history of hematological disorders. The physical examination and vital signs were within normal limits. Blood biochemistry and renal and hepatic function tests showed no anomaly either. Additional file 2 elaborates the detailed physical examination results.

On the 27th month (April 2018), a follow-up CT screening from the first surgery revealed vegetative tumor growth in the perineum requiring surgical management. In July 2018, the patient had transperineal pelvic tumor resection to stem the local recurrence of the tumor with the TNM staging of T3, N×, M1a (IVA). Histopathological examination showed a moderately differentiated adenocarcinoma and infiltration/metastasis associated with the invasion of the skin. All surgical margins were free of residual tumor. Immunohistochemical staining demonstrated positive expression of CDX-2, cytokeratin 20 (CK20) homeobox, and villin proteins, while cytokeratin 7 (CK7) protein demonstrated negative expression. First-line adjuvant chemotherapy consisting of irinotecan 240 mg/m^2^ + levoflofolate 150 mg + fluorouracil 0.6 g (intravenous) was initiated. Additionally, fluorouracil 0.9 g was administered to the patient as an infusion. The patient showed stable disease progression during the treatment, with an adverse reaction of Grade 3 gastrointestinal discomfort. However, during a follow-up CT screening in December 2018, a small inguinal nodule was found on the left side without any discomfort. By March 2019, the tubercle size increased gradually even with oral homeopathic treatment (cantharis capsule) by Heishi County Third People's Hospital (unknown specifics) with a poor prognosis. The further enlargement of the nodules called for another screening. CT scan indicated multiple metastatic tumors, rectal fistula, and enlargement of bilateral inguinal lymph nodes. Due to further enlargement of nodules with the formation of gall stones and enlarged inguinal lymph nodes, the patient received local bilateral inguinal radiography for bilateral inguinal lesions to a dose of 50 Gy in 25 fractions. Follow-up CT imaging showed recurrent metastasis, due to which following the subsequent modification of second-line chemotherapeutic regimen to bevacizumab 400 mg + oxaliplatin 200 mg + capecitabine 1.5 g (twice daily) was given for 14 days. However, due to adverse gastrointestinal reaction (Grade > 3) caused by due to oral capecitabine, the capecitabine dose was reduced to 1 g, this modified second-line chemotherapeutic regimen was led to an interruption in chemo regimen, following which bevacizumab 400 mg + oxaliplatin 200 mg + capecitabine 1 g (twice daily) was started and given for a single cycle in January 2020. The multiple rounds of chemotherapy showed partial response with recurrence of multiple nodules and masses. A follow-up CT imaging in March 2020 showed disease progression, additionally presented nodule formation in the left upper lobe and right lower lobe, with an iso-dense nodule. The TNM status at this instance was T3, N×, M1b (IVB).

**Fig. 1:**
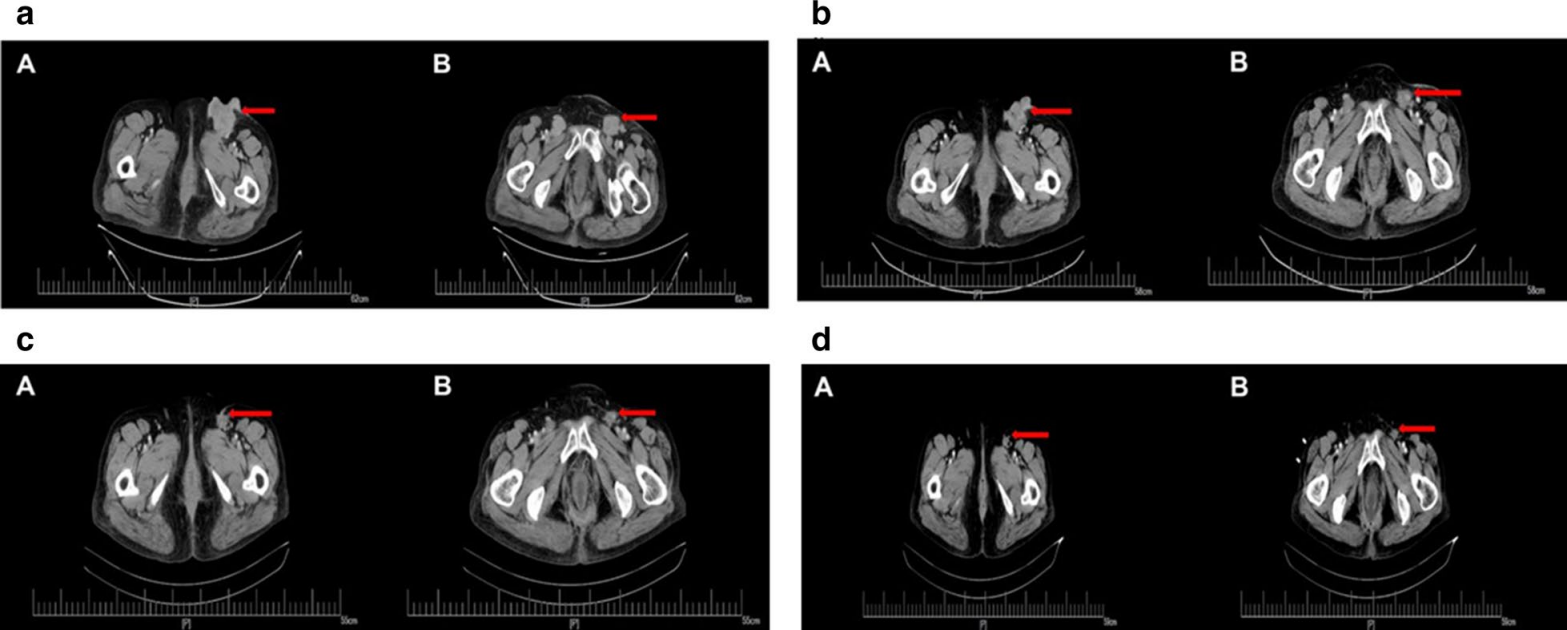
CT: pelvic cavity to assess disease status and progression. CT image of pelvic cavity to assess disease status before starting treatment with regorafenib and sintilimab (**a** Arrows indicate metastatic focus before treatment). CT image of pelvic cavity to assess disease progression with regorafenib and sintilimab treatment before treatment (**b**–**d** Arrows indicate metastatic focus shows that the tumor size has decreased during treatment duration)

Further, the patient tested positive for hepatic C virus, following which an oral administration of Jisandai (Bantongsha) that is sofosbuvir 400 mg and velpatasvir 100 mg as a composite tablet once a day was administered. Adverse effects included tooth pain caused by gingivitis, which was cured by oral administration of ornidazole 500 mg twice daily. Following recurrence of rectal adenocarcinoma, regorafenib was administered at 120 mg once daily combined with sintilimab 200 mg five times a day, and the patient's progress was followed up (as an out-patient once every week). Eventually, the sintilimab dosage was tapered to 100 mg four times a day due to economic reasons. CT scans of the chest and abdomen suggested that treatment achieved a complete response (CR). During the last follow-up in April 2020, re-examination by CT imaging (Fig. 1a–d) showed that the bilateral inguinal area had multiple nodules and masses, some of which were slightly smaller than the previous assessment. Follow-up CT imaging showed the absence of the frontal lobe iso-dense nodule that was present before. There were no severe adverse events, and the patient presented good overall performance status. The patient also received prophylactic treatment for hepatitis C. Additional file 3 briefly outlines the reports of the medical imaging history. The clinical effect of regorafenib plus sintilimab was classified as CR according to Response Evaluation Criteria in Solid Tumors (RECIST) version 1.1 criteria, and the patient exhibited no tumor progression. CT imaging could not identify the presence of metastasis. The predictors of efficacy of regorafenib combined with sintilimab remain to be further analyzed.

## Discussion and conclusion

The recent emergence of nanomedicine and ICIs such as PD-1 has revolutionized the treatment, hypothesized to improve the quality of life and extend the survival time with CRC. The PD-1 inhibitor sintilimab is a promising new drug with pharmacokinetic properties similar to nivolumab. Compared with nivolumab and pembrolizumab, two well-studied PD-1 inhibitors approved by China National Medical Products Administration and the US FDA, sintilimab has a similar antitumor effect, a better safety profile, and obvious pharmacoeconomic advantages [14]. However, even with the advancement of therapies, MSS tumors are challenging to treat as they have a low rate of mutations and consequently lack adequate immune activation [16]. In these settings, regorafenib has demonstrated the ability to reduce tumor-associated macrophages by blocking the Colony stimulating factor 1 (CSF-1) receptors and is hypothesized to confer sensitivity to tumors [17]. Also, PD-L1 expression can be blocked while maintaining Major histocompatibility complex class I (MHC-1) expression [17]. Therefore, the addition of immunotherapeutic agents hypothesized the alteration of the tumor microenvironment and enhancement of the intratumoral immune response to ICIs. Moreover, the immunotherapeutic agents might also elicit synergistic effects when combined with antiangiogenic agents, chemotherapy, local ablative therapies, and hepatic arterial infusions in CRC [18].

In a recent Japanese phase Ib /dose-expansion trial, Fukuoka et al. found an improved response with the combination of regorafenib and nivolumab showed an improved response in MSS CRC tumors, with overall response rate (ORR) 5.5% with regorafenib 80 mg and 36.0% with 120 mg [19]. In this study, the median progression-free survival PFS of CRC was 7.9 months, median overall survival (OS) was unachieved, and the expected 1-year OS rate was 68% [19]. In contrast, the data presented by Kim et al. at European Society for Medical Oncology Asia Virtual Congress 2020 showed slightly different results [20]. Out of 21 patients evaluated, only one had a partial response, while 67% of patients had stable disease with a median PFS of 4.3 months, and median OS of 11 months [20]. The discrepancy of US trial data might be due to the ethnic differences and variation in inclusion criteria [20]. However, both the trial results demonstrated improved tumor response rates and PFS with a combination of regorafenib and PD-1 inhibitor in CRC. In concordance with the data obtained from two clinical trials, the results from this case study also indicate that the combination therapy with regorafenib and PD-1 inhibitor, sintilimab improved the clinical outcome of a patient with refractory CRC previously treated with multiple lines of chemotherapy.

A retrospective study conducted in patients with MSS metastatic CRC receiving an anti-PD-1 inhibitor (nivolumab/pembrolizumab/camrelizumab/sintilimab/toripalimab) combined with regorafenib as third- or further line of treatment showed the median PFS of 3.1 months (95% CI 2.32–3.89) with 78.3% achieving stable disease. Treatment-related Grade 3 adverse event was seen in 21.7% of patients [21].

Also, Zhang et al. showed that the sequential treatment with regorafenib followed by sintilimab in a Sorafenib-Refractory hepatocellular carcinoma patient achieved a CR in their case report [22]. The significant highlight of the present case study is that it demonstrates that the combination of regorafenib plus sintilimab administered in an MSS stable CRC patient refractory to previous lines of treatment achieved CR and stable disease culminating antitumor activity. To our knowledge, this is the only case report by far that investigates the clinical outcomes of third-line regorafenib and sintilimab combination in patients with CRC.

In China, 35–40% of patients with CRC harbor KRAS mutations that more commonly occur in codon 12 (79.1%) and codon 13 (20.4%) in exon 2 [23]. The patient in this case report also detected KRAS codon 12 and 13 mutations, although no study has concretely established KRAS mutations as an independent prognostic factor in CRC with any significant correlation [6]. Moreover, the histopathology and clinical features of this tumor include multiple lines of treatment suggesting a poor prognosis. A possible explanation of this augmented clinical effect could be the previously mentioned alteration in the microenvironment [3]. Thus, the lack of generalizability and the retrospective nature must be accounted for while scrutinizing this study. Many ongoing trials such as Regosinti [NCT04745130], APICAL-CR [NCT04745130], and a real-world study [NCT04771715] are evaluating the clinical outcomes of combining oral tyrosine kinase inhibitor and PD-1 inhibitors in CRC.

In conclusion, this case report has demonstrated favorable clinical outcomes of the novel PD-1 inhibitor, sintilimab in combination with regorafenib in a patient with refractory MSS CRC as a third-line treatment option. The patient achieved stable disease and CR with no incidence of adverse reactions above Grade 3 with the combination therapy of regorafenib and sintilimab. We recommend that the detailed molecular analysis of the tumor microenvironment may provide additional insights into the long-term survival with regorafenib and sintilimab combination therapy, hence warranting an unmet need for further clinical studies.

## Supplementary Information

**Additional file 1.** Patient images for disease progression (1a, 1b, 1c, and 1d).

**Additional file 2.** Detailed patient history.

**Additional file 3.** Detailed report of CT scan during follow-up period.

## Data Availability

The datasets used and/or analyzed during the current study are available from the corresponding author on reasonable request.
