# Supplementary material for: Long-term survival of a patient with microsatellite-stable refractory colorectal cancer with regorafenib and PD-1 inhibitor sintilimab: a case report and review of literature
Source: BMC Gastroenterol. 2021 Oct 23;21:399. doi: 10.1186/s12876-021-01950-y (PMC8542310; doi:10.1186/s12876-021-01950-y)

**Additional file 1a:** Patient images for disease progression


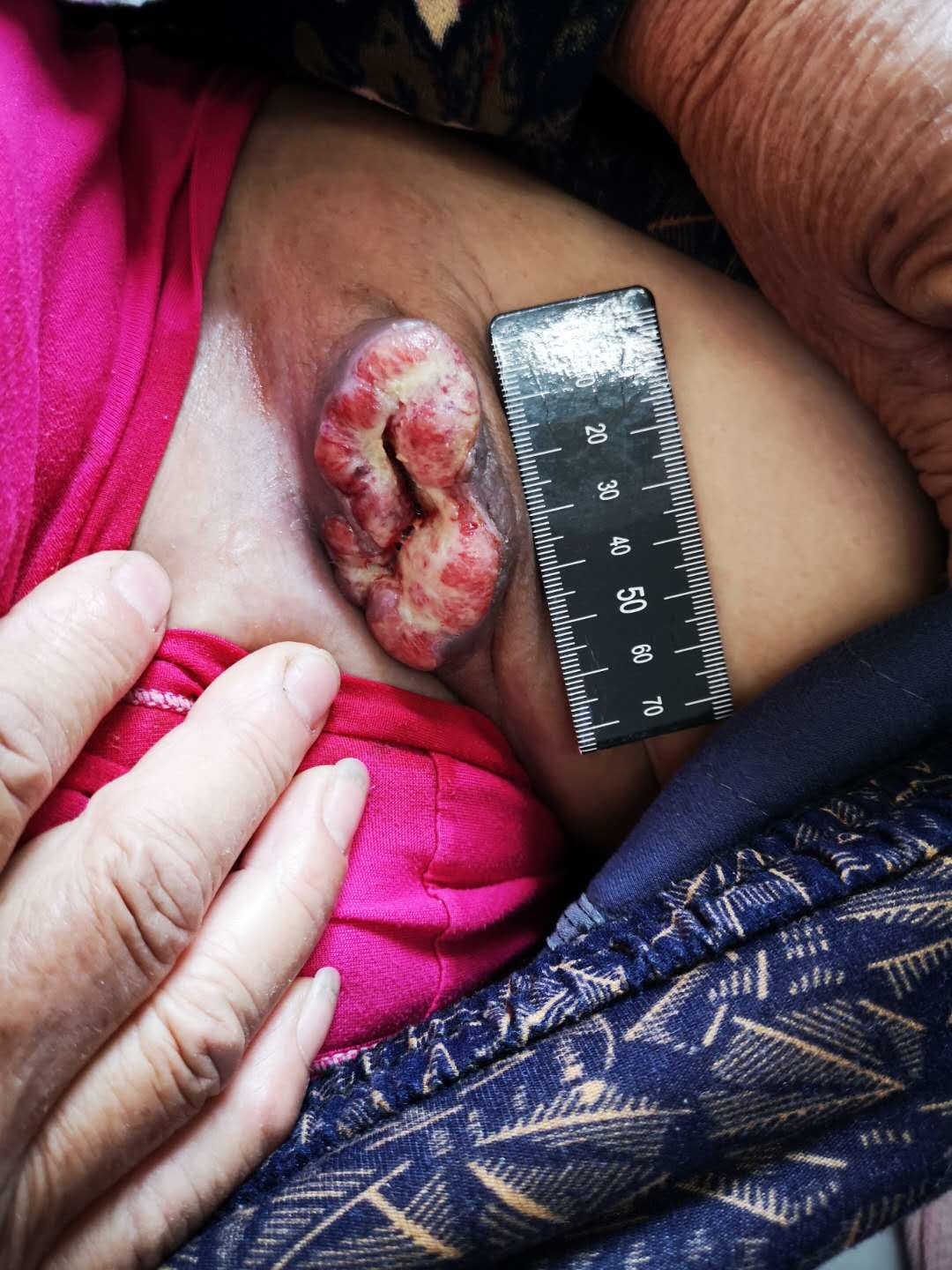

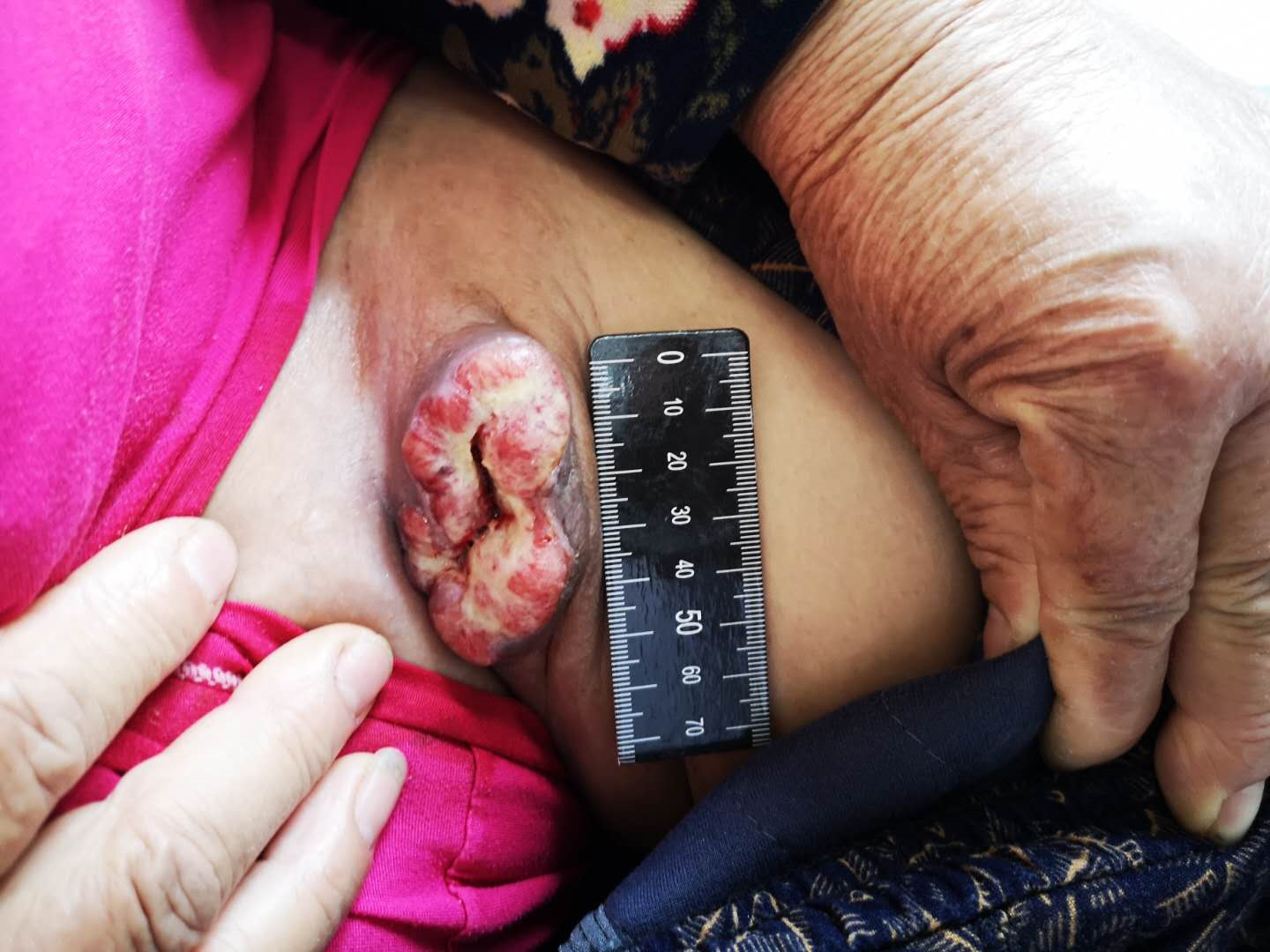

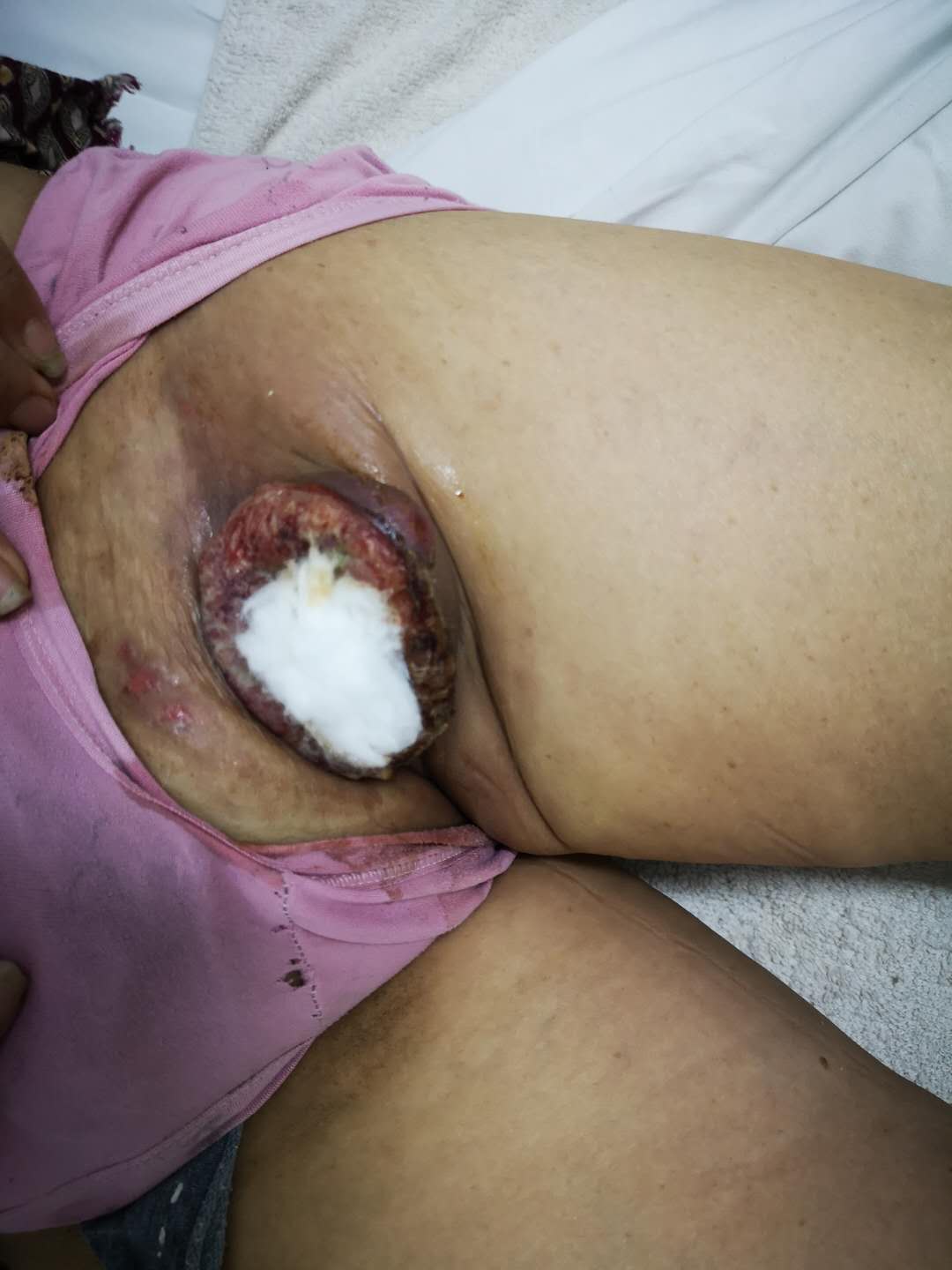

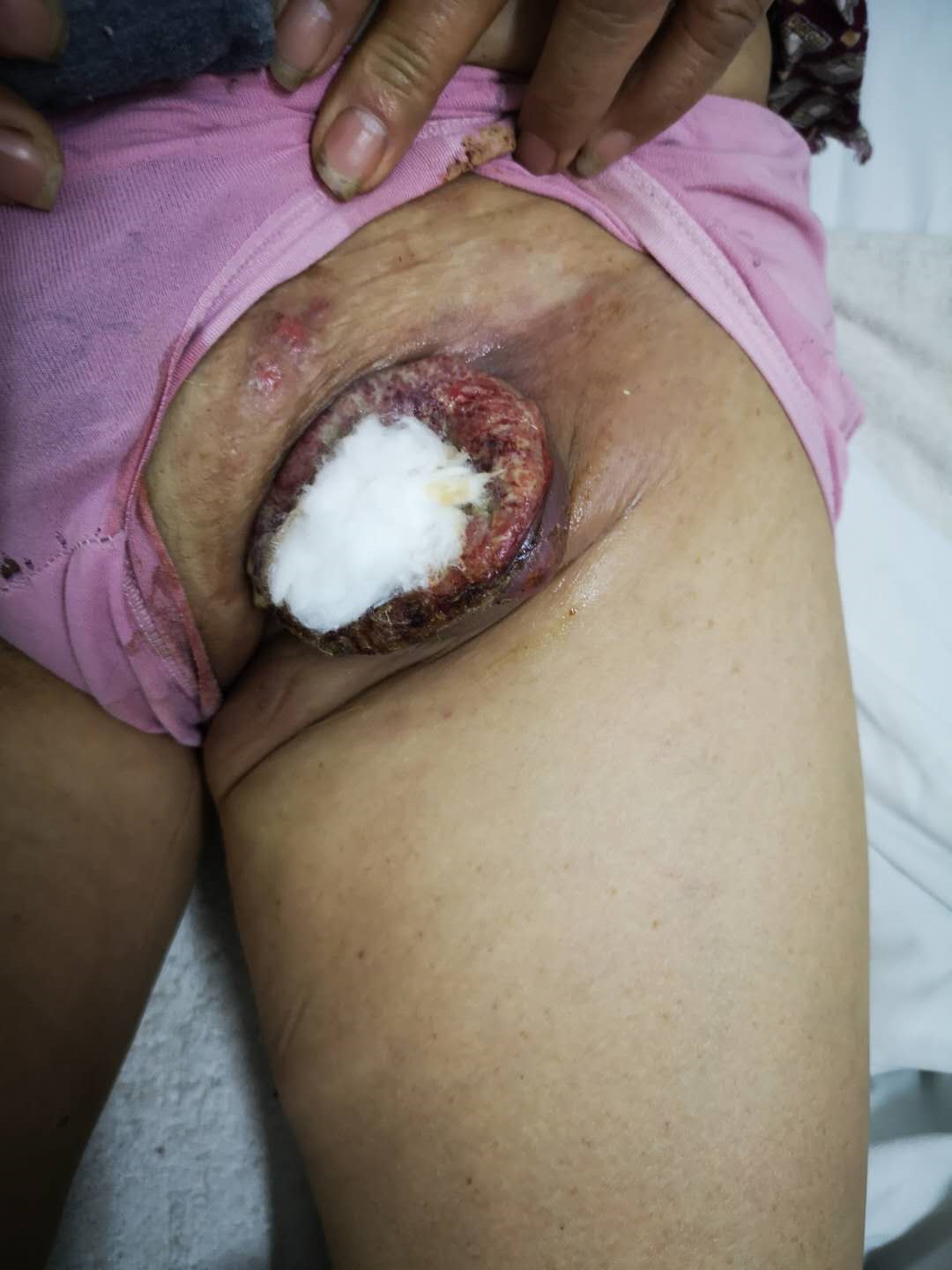


**Additional file 1b:** Patient images for disease progression


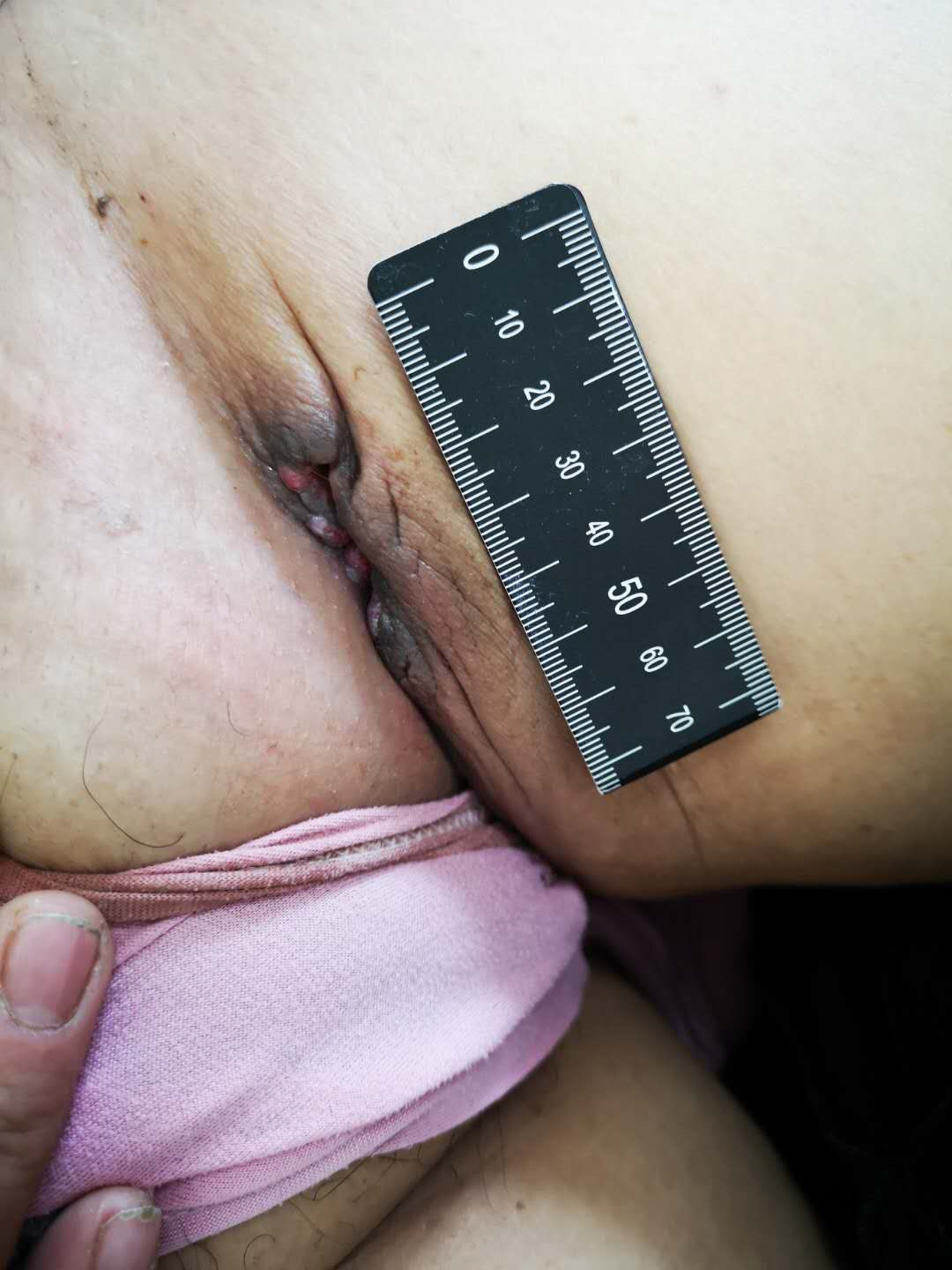

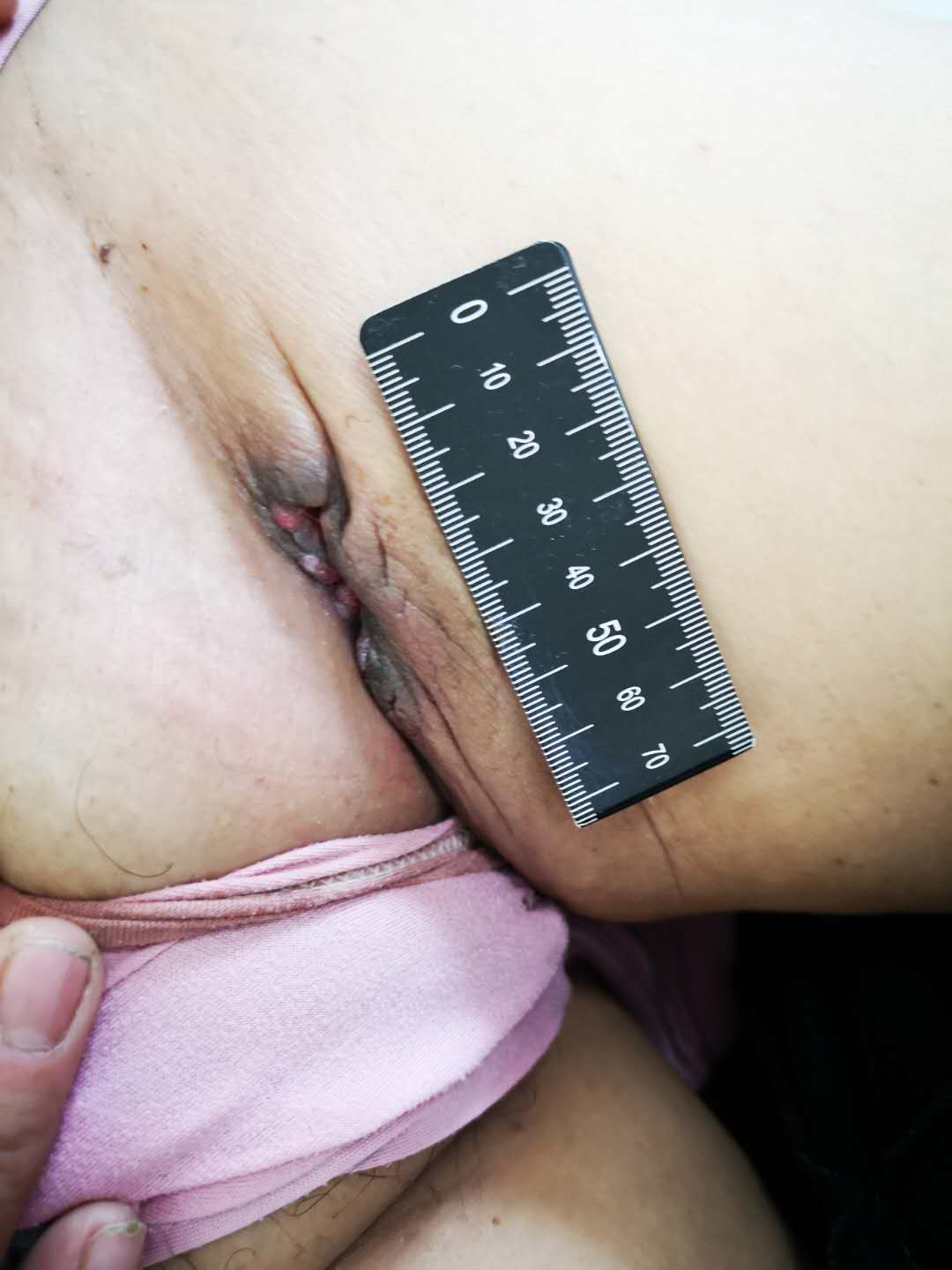

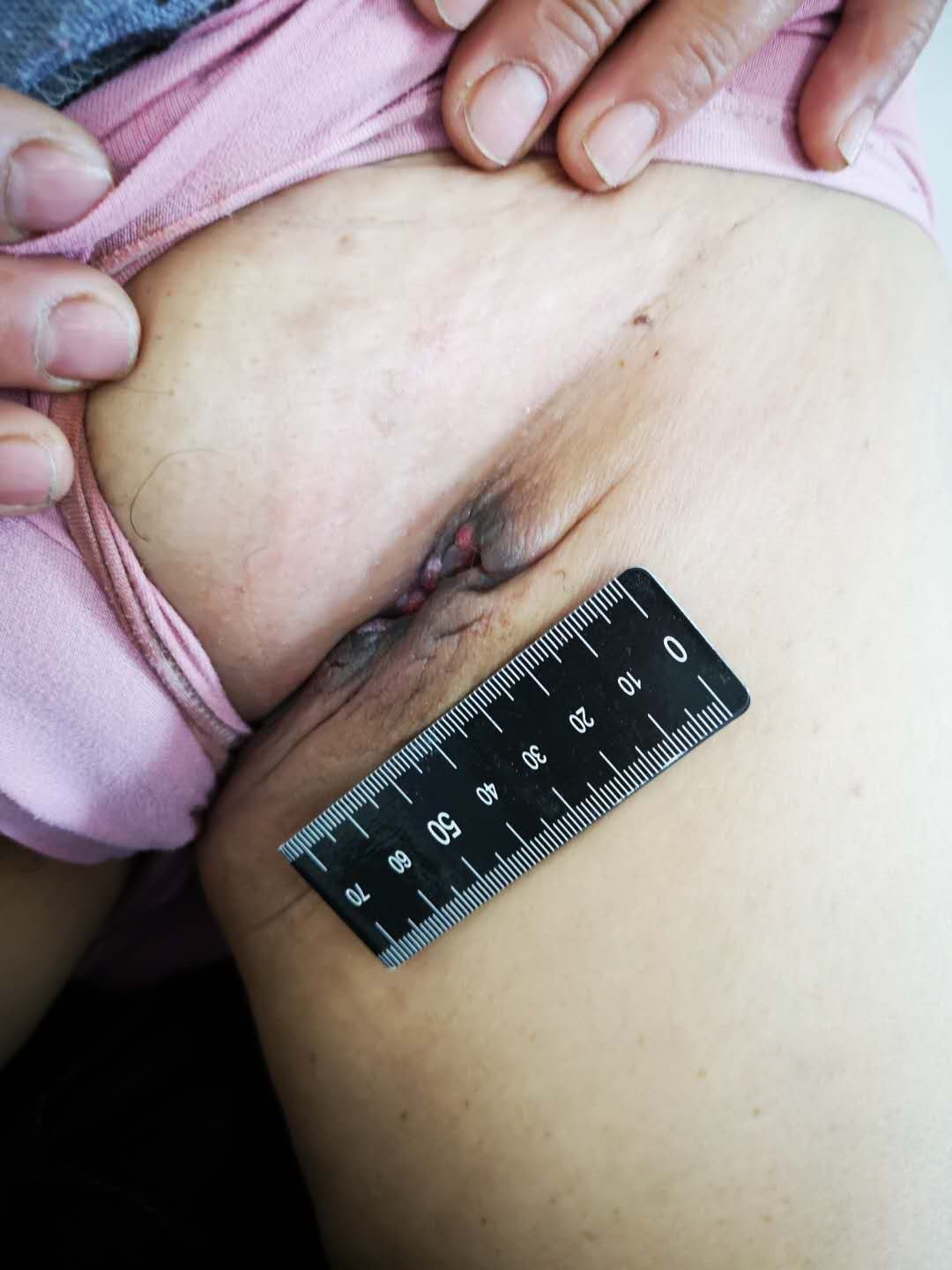

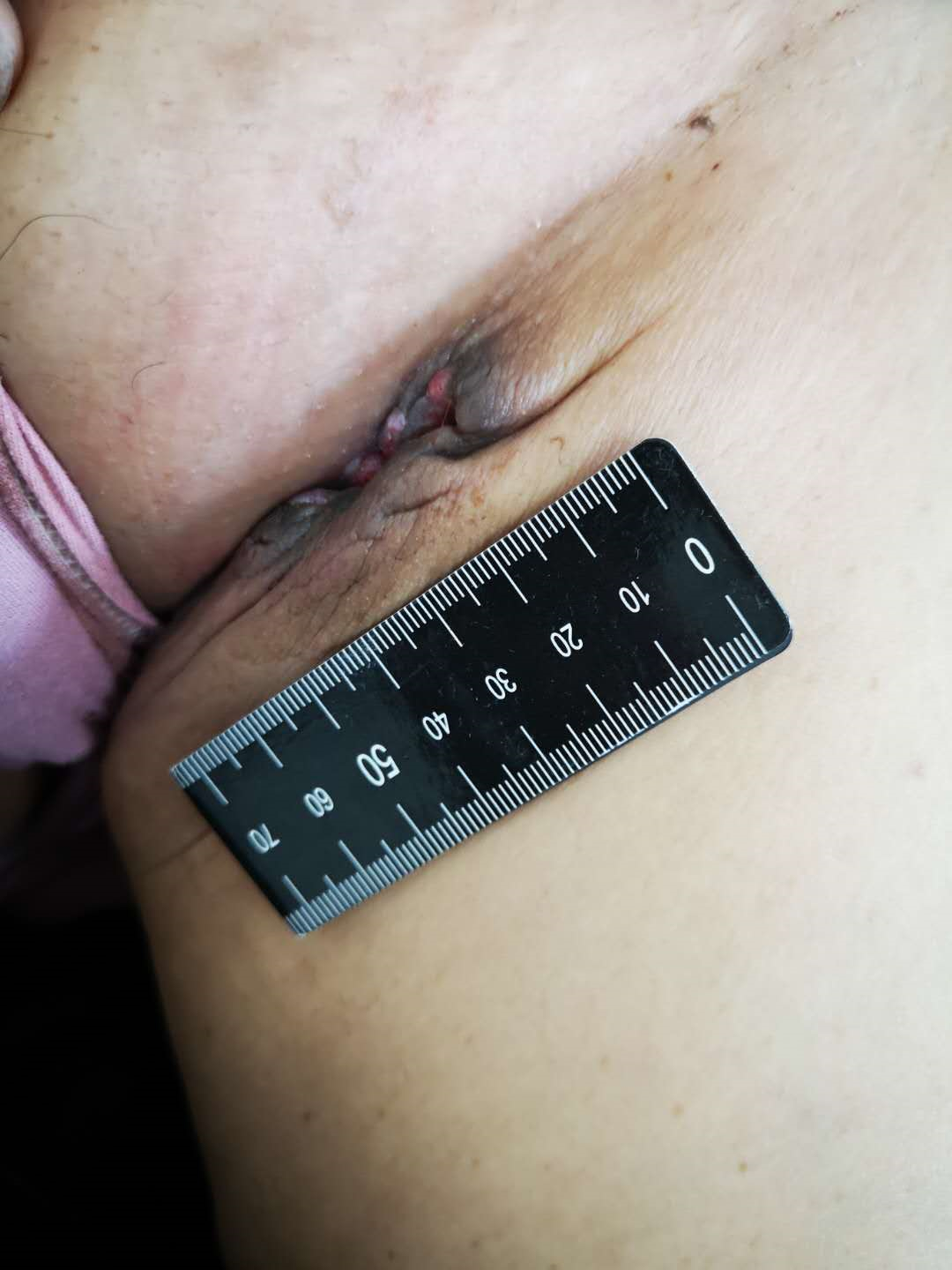


**Additional file 1c:** Patient images for disease progression


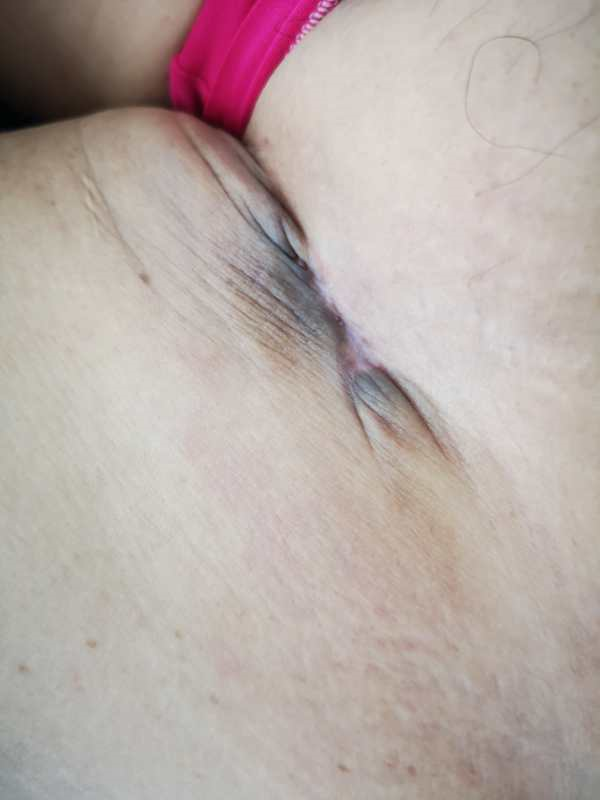

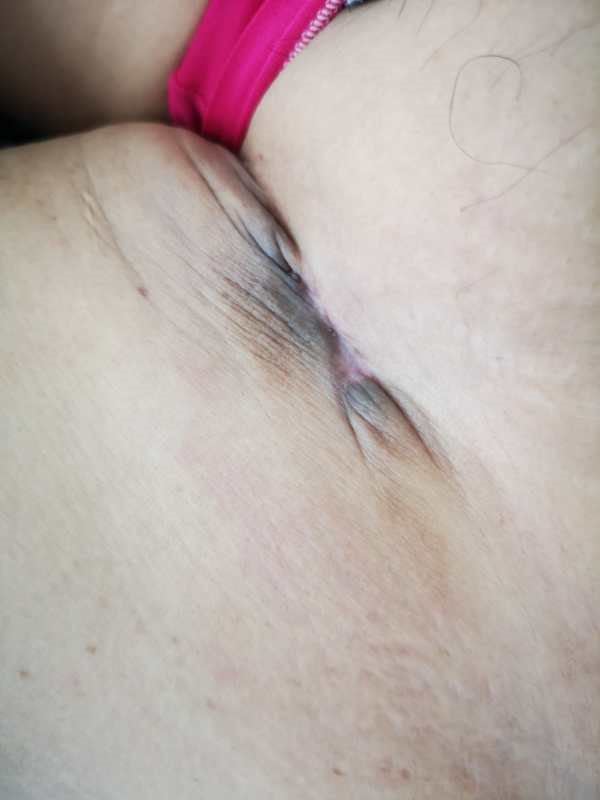

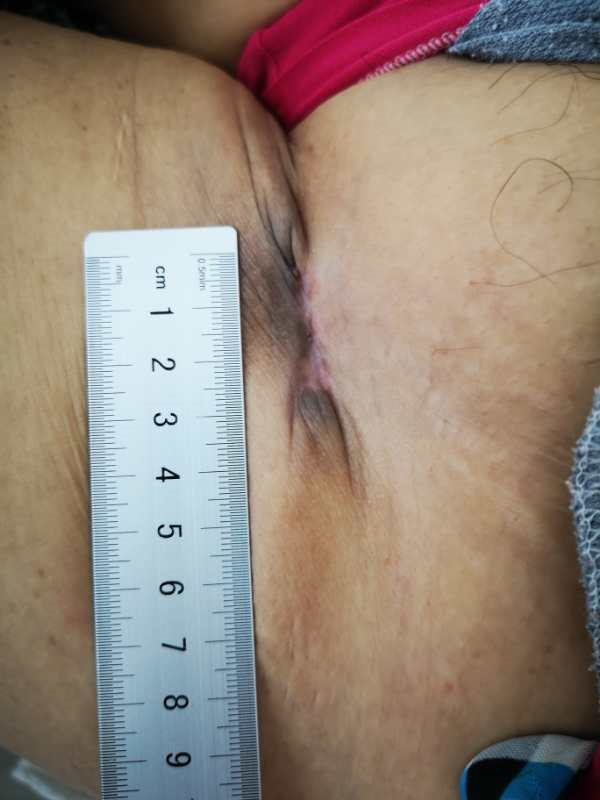


**Additional file 1d:** Patient images for disease progression


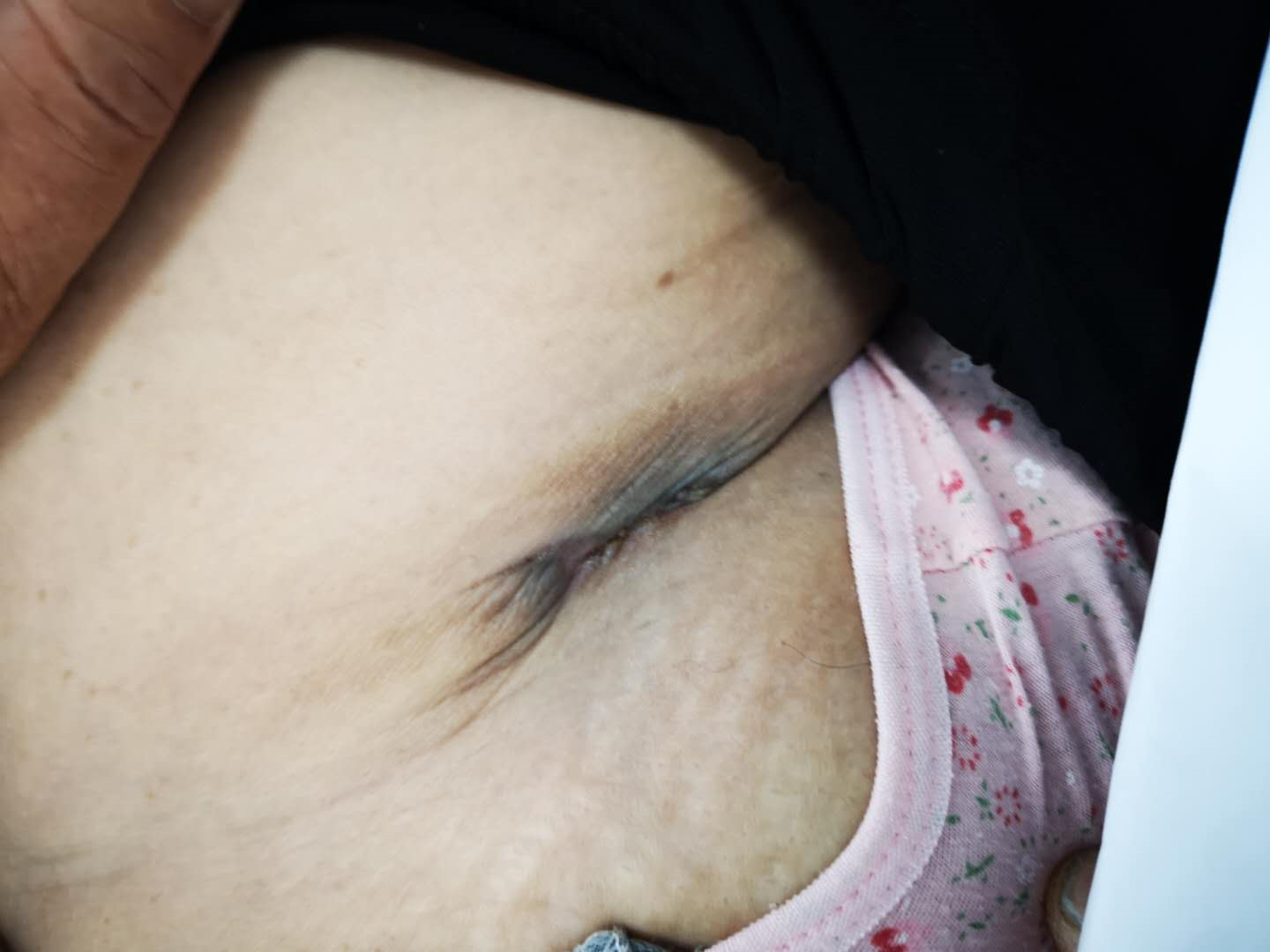

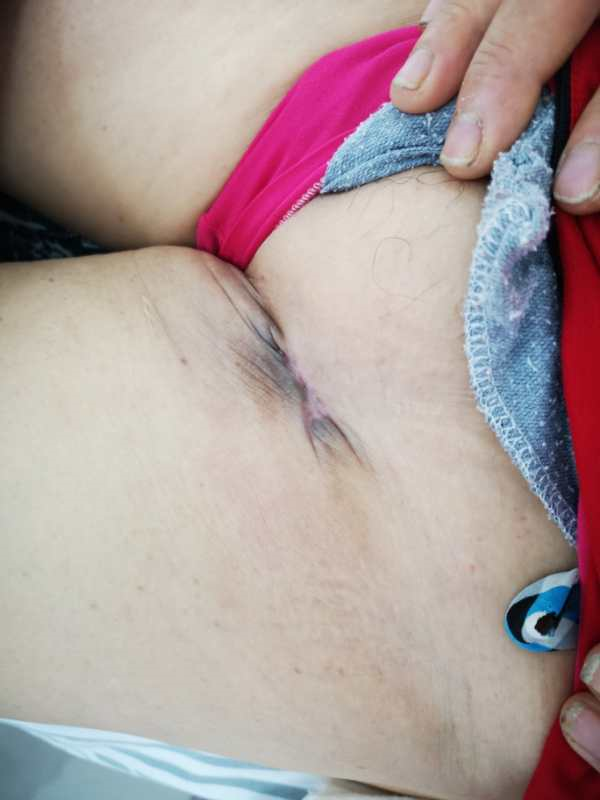


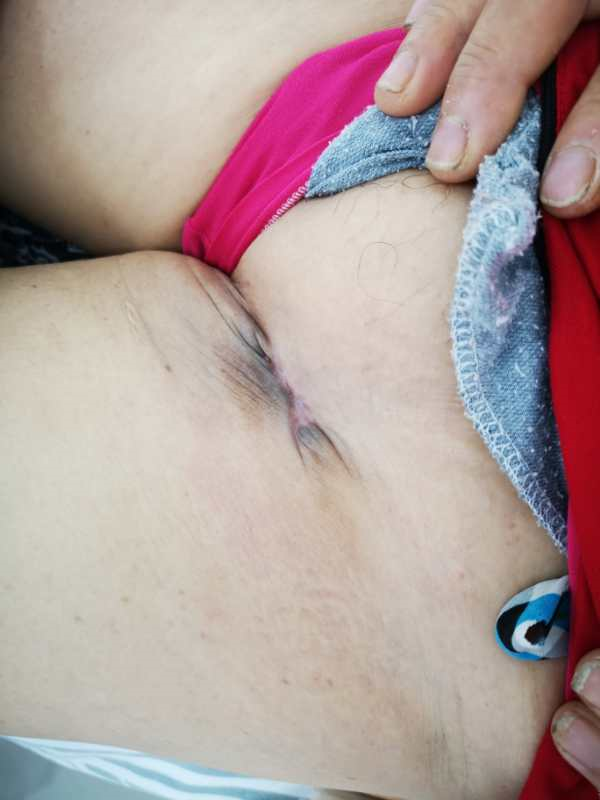

Supplement: Supplementary file 1 — Additional file 1. Patient images for disease progression (1a, 1b, 1c, and 1d). [file 12876_2021_1950_MOESM1_ESM.docx]
