# Supplementary material for: Long-term survival of a patient with microsatellite-stable refractory colorectal cancer with regorafenib and PD-1 inhibitor sintilimab: a case report and review of literature
Source: BMC Gastroenterol. 2021 Oct 23;21:399. doi: 10.1186/s12876-021-01950-y (PMC8542310; doi:10.1186/s12876-021-01950-y)
Supplement: Supplementary file 2 — Additional file 2. Detailed patient history. [file 12876_2021_1950_MOESM2_ESM.docx]

**Additional file 2**: Detailed patient history

| Details | History |
| --- | --- |
| General | 24-year-old married, husband in good health; 2 sons and 1 daughter, all healthy.  Menstrual history: menarche 13 years old, 3-5/28-30 years old, 50 years old. Regular menstrual cycle, moderate menstrual volume and normal color.  Family history: the parents died for unknown reasons; one elder brother and one elder sister, one younger brother and one younger sister were in good health; family genetic history was denied. |
| Physical Examination | Temperature: 36.5, Blood pressure: 120/80mmHg |
| Head | No deformity, tenderness, mass, eyelid edema, normal conjunctiva, normal eyeball, no yellow stain of sclera, round pupil, normal light reflex, no abnormal secretion of external auditory canal, no tenderness of mastoid, no hearing impairment. The sense of smell is normal. There was no cyanosis on the lips and normal oral mucosa. The tongue coating was normal, no deviation or tremor was found in the tongue extension, the gingiva was normal, the pharyngeal mucosa was normal, and the tonsil was not enlarged. |
| Neck | Soft neck without resistance, normal carotid pulse, normal jugular vein, trachea in the middle, negative hepatic jugular vein reflux sign, normal thyroid, no tenderness, tremor, vascular murmur |
| Chest | The thorax is normal, the sternum has no percussion pain, and the breast is normal and symmetrical. The respiratory movement was normal, the breathing was regular, the intercostal space was normal, and the speech fibrillation was normal. Percussion was used to clear the sound. The breath sounds of both lungs were clear without pleural friction. There was no protrusion in the precordial area, and the apical pulsation was normal. During percussion, the relative murmur boundary of the heart was as shown in the figure below. The heart rate was 80 beats/min and the rhythm was regular. There was no murmur in the auscultation area of each valve, and there was no pericardial fricative sound |
| Abdomen | Flat abdomen, no varicose veins, soft abdomen, no tenderness, rebound pain, abdominal mass. The liver was not touched, the spleen was not touched, Murphy's sign was negative, the kidney had no percussion pain, no mobile dullness. Bowel sounds were normal, 4 times/minute. |
| Anus and genitalia | Anus and external genitalia were not checked |
| Nervous system | Muscle strength and muscle tension of limbs were normal, bilateral biceps and triceps tendon reflex was normal, bilateral knee and Achilles tendon reflex was normal, bilateral Babinski sign was negative. |
