# Supplementary material for: Long-term survival of a patient with microsatellite-stable refractory colorectal cancer with regorafenib and PD-1 inhibitor sintilimab: a case report and review of literature
Source: BMC Gastroenterol. 2021 Oct 23;21:399. doi: 10.1186/s12876-021-01950-y (PMC8542310; doi:10.1186/s12876-021-01950-y)
Supplement: Supplementary file 3 — Additional file 3. Detailed report of CT scan during follow-up period. [file 12876_2021_1950_MOESM3_ESM.docx]

**Additional file 3**: Detailed report of Computerized Tomography (CT-Scan) during
 follow up period

| Post-operative pathology | Examination Details |
| --- | --- |
| January 2016 | Ulcerative moderately differentiated adenocarcinoma (rectum and part of sigmoid colon) invaded adventitia. (two cutting edges) clean. (peri intestinal lymph node) 0/6. (para intestinal lymph nodes) 0/6. (additional paravascular lymph node sent) 0/7. Gene detection (2016.1.19): 1. KRAS gene: the detection range of codons 12 and 13 is wild type. 2. MSI detection: microsatellite stability (MSS). |
| July 25, 2018 | After rectal cancer surgery and chemotherapy): perineum) tissue showed moderately differentiated adenocarcinoma infiltration/metastasis, invasion of skin, combined with the history and morphology, consistent with intestinal origin.(the upper, lower, left, right and rear cutting edges are sent to clean). Immunohistochemistry: CDX-2+, CK7-, CK20+, villin+. |
| October, 2018 | Pelvic implant metastasis changes. The original pelvic effusion was not clearly shown. 3. The bladder wall is not uniform and slightly thickened, and the change is not obvious. Low density of right kidney, cyst considered, similar to the former. 8. Nodular shadow in the upper lobe of the right lung, which may be inflammatory, should be followed up. Gallstone, slightly more than before. |
| March 2020 | Post-operative changes in rectal cancer. 2. The amount of effusion in the anterior sacral rectum was less than that in the anterior. 3. Multiple nodules and masses in bilateral inguinal area, larger than before, should be considered for metastasis. 4. Small lymph nodes were scattered between the liver and stomach, retroperitoneum, mediastinum and the right cardiac diaphragmatic angle. 5. Fatty liver, better than before. 6. Low density of right kidney, cyst considered, similar to the former. 7. Gallstone, slightly more than before. 8. Small nodule in the left adrenal junction, adenoma possible, follow up. 9. The former nodule shadow in the upper lobe of the right lung, which may be inflammatory, is not clear. 10. The nodules in the left upper lobe and right lower lobe were similar in some cases and new in some cases. 11. There was a little inflammation in the middle lobe of the right lung, which was similar to the former, and a few cords in the upper lobe of the left lung. 12. Inflammation of bilateral maxillary sinus, ethmoid sinus and right sphenoid sinus. 13. Scattered lymph nodes in bilateral neck. 14. Suspicious isodense nodule in left frontal lobe,. |
